# Birds of Two Oceans? Trans-Andean and Divergent Migration of Black Skimmers (*Rynchops niger cinerascens*) from the Peruvian Amazon

**DOI:** 10.1371/journal.pone.0144994

**Published:** 2016-01-13

**Authors:** Lisa C. Davenport, Katharine S. Goodenough, Torbjørn Haugaasen

**Affiliations:** 1 Florida Museum of Natural History, Department of Natural History, University of Florida, Gainesville, Florida, United States of America; 2 Center for Tropical Conservation, Duke University, Durham, North Carolina, United States of America; 3 Oklahoma Biological Survey, University of Oklahoma, Norman, Oklahoma, United States of America; 4 Department of Ecology, Norwegian University of Life Sciences, Ås, Norway; INIBIOMA (Universidad Nacional del Comahue-CONICET), ARGENTINA

## Abstract

Seasonal flooding compels some birds that breed in aquatic habitats in Amazonia to undertake annual migrations, yet we know little about how the complex landscape of the Amazon region is used seasonally by these species. The possibility of trans-Andes migration for Amazonian breeding birds has largely been discounted given the high geographic barrier posed by the Andean Cordillera and the desert habitat along much of the Pacific Coast. Here we demonstrate a trans-Andes route for Black Skimmers (*Rynchops niger cinerascens)* breeding on the Manu River (in the lowlands of Manu National Park, Perú), as well as divergent movement patterns both regionally and across the continent. Of eight skimmers tracked with satellite telemetry, three provided data on their outbound migrations, with two crossing the high Peruvian Andes to the Pacific. A third traveled over 1800 km to the southeast before transmissions ended in eastern Paraguay. One of the two trans-Andean migrants demonstrated a full round-trip migration back to its tagging location after traveling down the Pacific Coast from latitude 9° South to latitude 37° S, spending the austral summer in the Gulf of Arauco, Chile. This is the first documentation of a trans-Andes migration observed for any bird breeding in lowland Amazonia. To our knowledge, this research also documents the first example of a tropical-breeding waterbird migrating out of the tropics to spend the non-breeding season in the temperate summer, this being the reverse pattern with respect to seasonality for austral migrants in general.

## Introduction

The Amazon Basin is a world driven by water, controlled by a geographic situation that creates a complex hydrology, with important consequences for aquatic life [1]. Annual flooding dramatically alters the landscape each year, reconnecting rivers with oxbow lakes, flooding large expanses of forest, and completely submerging river bars for weeks or months [2,3]. Straddling the equator along a long East-West axis, the Basin integrates rainfall from the eastern slopes of the Andes with rainfall from northern and southern tributaries that experience peak volumes offset by six months [3]. Peak rainfall may coincide with low water at some locales, and both low and high water conditions may adjoin where tributaries on opposite schedules converge. In high water conditions, fish disperse widely to feed on forest-derived resources, while in low water, fish concentrate in the main river course or remaining backwaters, altering feeding opportunities for piscivorous mammals and birds [4]. In addition to varying freshwater conditions, the coasts of South America offer some of the most productive and biodiverse marine habitats on earth, with productivity driven by the Humboldt Current’s rich upwelling zones on the Pacific, and highly productive regions at the mouths of major rivers on the Atlantic [5]. Breeding birds tied to the Amazon’s aquatic cycles are thus presented with a complicated temporal mosaic of habitat and feeding conditions and should show movement patterns that optimize local and regional opportunities.

Information on bird migration within South America is still deficient, despite considerable interest in the use of South American habitats by many Neotropical migrants [6–8]. Work on intra-tropical migrants is particularly scant, and for Neotropical waterbirds, many of which are migratory [7,9], most species’ migration patterns are still unknown, even in the region’s most important wetlands [10]. Studies that track individuals’ movements provide ideal data on time spent in different locations through entire annual cycles, but tracking has rarely been used with Amazonian breeding birds. Previous tracking work in South America has focused more on terrestrial or Andean species [11–15] or seabirds [16], with only the Andean condor (*Vultur gryphus*), ever demonstrating movement patterns between lowland and Andean habitats [15]. Examples of studies on waterbird movements in Australia [17], Africa [18–20] and Asia [21] provide insight into patterns of avian migration in these areas, although cross-continental comparisons have so far allowed for few generalizations [6,22] and provide little predictive power for Amazonia. Research with Black Skimmers (*Rynchops niger cinerascens*) reported here, combined with earlier work from the same site on Orinoco Geese [23] therefore comprises some of the first work to shed light on heretofore unknown migratory routes of Amazonia’s waterbirds.

Skimmers (*Rynchops spp*.) are piscivorous waterbirds from the Americas, India and Africa, notable for their specialized bill and flight mechanics that allow them to catch fish on the wing using only tactile cues [24]. Tropical skimmers, such as the African and Indian skimmers (*Rynchops flavirostris and Rynchops albicollis*, respectively) are predominantly riverine species [25–26] while the North American subspecies of the Black Skimmer (*Rynchops niger niger*) primarily inhabits marine environments, although inland breeding is known [27]. Black Skimmers in South America breed on riverine sandbars during low-water periods when point bars are exposed [28–29]. Two South American subspecies are recognized: *Rynchops niger cinerascens* from northern and western South America; and *Rynchops niger intercedens* from Eastern Brazil to NE Argentina [30]. Although both subspecies use coastal areas in the non-breeding season, concentrations only rarely include *R*. *n*. *cinerascens* on the Atlantic coast or *R*. *n*. *intercedens* on the Pacific coast ([31] and F. Schmitt, pers. comm.).

Black Skimmers appear seasonally in large numbers on both the Pacific and Atlantic coasts of South America, being most common September to April [28, 31–33]. The timing coincides with the end of the breeding season in the southern Amazon and the onset of heavy rains that raise river levels and flood breeding beaches. The possibility that Amazonian skimmers were appearing on the Pacific coast of Peru was discounted by Hughes [32] who surmised that Black Skimmers arriving each year in Mollendo (Pacific Coast of Peru, latitude -17.02; longitude -72.02) were likely from North America. Trans-Andes migration by Amazonian-breeding birds has generally been deemed unlikely given the high geographic barrier posed by the Andean Cordillera, and the unsuitable desert habitat along much of the Pacific Coast. Yet, scattered wetlands and sheltered bays which dot the Pacific Coast of Peru and Chile share several species with lowland Amazonia, for example herons (Family Ardeidae), shorebirds (Family Charadridae) and skimmers. In addition, high Andean lakes have been documented to be used by some species of migratory waterbirds, including Nearctic migrant shorebirds, although the detailed routes used by these birds either to cross or to parallel the Andes during migration is unknown [34]. Nevertheless, high-altitude migrations are known from other waterbirds such as the Arctic Tern *Sterna paradisea* [35] and geese [36–37], and a number of shorebirds regularly appear in Andean habitats above 3,000 m, suggesting that high elevations and low temperatures are no obstacle to certain species. Field observations of large groups of skimmers post-breeding (November) in the Manu region of Atalaya, at the base of the Andes and in a region where they are not documented to breed, suggest that congregations of skimmers may stage collective crossings over the Andes from this locality (LCD and J Terborgh, pers. obs.).

Our goal in this study was to test the hypothesis that Manu River skimmers cross the Andes to the Pacific Coast or, alternatively, seek suitable water conditions by crossing the Equator to northern Amazonia or moving elsewhere in tropical basins where the conditions are reversed with respect to river levels.

## Methods

### Study Site

The Manu River is a meandering white-water river at the base of the Peruvian Andes that fluctuates seasonally, exposing extensive (up to 2 km long) point bars (beaches) during its dry season (May–October) [3,38]. The entirety of its watershed is protected within the Manu National Park [38]. Point bars exposed during the dry season provide important seasonal nesting habitat for both turtles and a suite of nesting birds, including Black Skimmers [29].

### Capture and PTT Deployment

**Fig 1:**
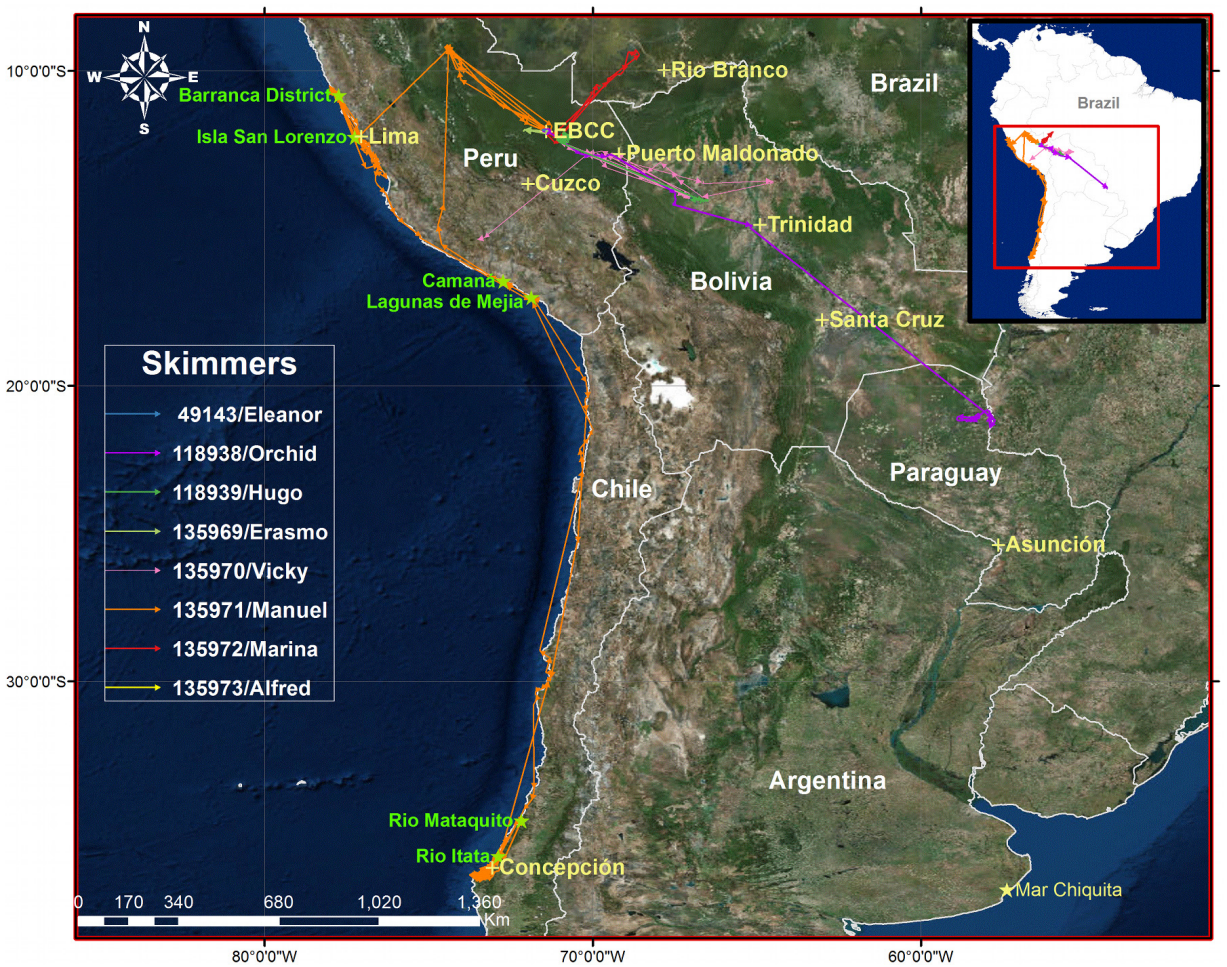
Long-distance movements of Black Skimmers tagged in Manu National Park, Perú, 2012–2014. Tracks of 8 skimmers tagged in Manu National Park with arrows denoting received locations and direction of travel. Pacific coast stopovers used by Manuel (orange track) for >48 h noted with green stars. Created with ESRI ArcGIS 10.2.2 software. Basemap data sources include: Esri, DigitalGlobe, GeoEye, Earthstar Geographics, CNES/Airbus DS, USDA, USGS, AEX, Getmapping, Aerogrid, IGN, IGP, swisstopo, and the GIS User Community.

**Fig 2:**
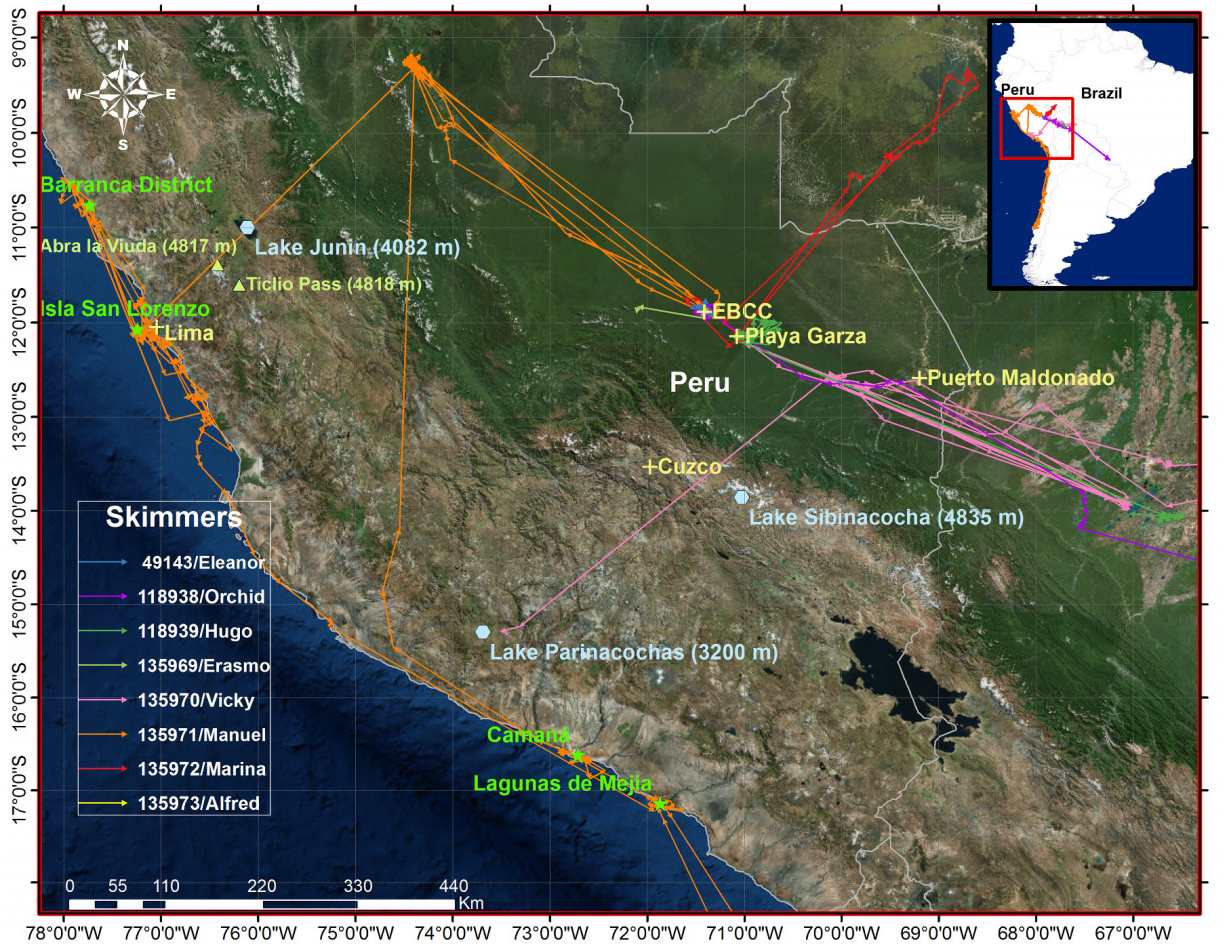
Trans-Andes migration routes used by two Manu Black Skimmers. Routes of Manuel (orange) and Vicky (pink) depict the varied routes used for the 3 Andean crossings tracked for two skimmers. Created with ESRI ArcGIS 10.2.2 software. Basemap data sources include: DigitalGlobe, GeoEye, Earthstar Geographics, CNES/Airbus DS, USDA, USGS, AEX, Getmapping, Aerogrid, IGN, IGP, swisstopo, and the GIS User Community.

**Fig 3:**
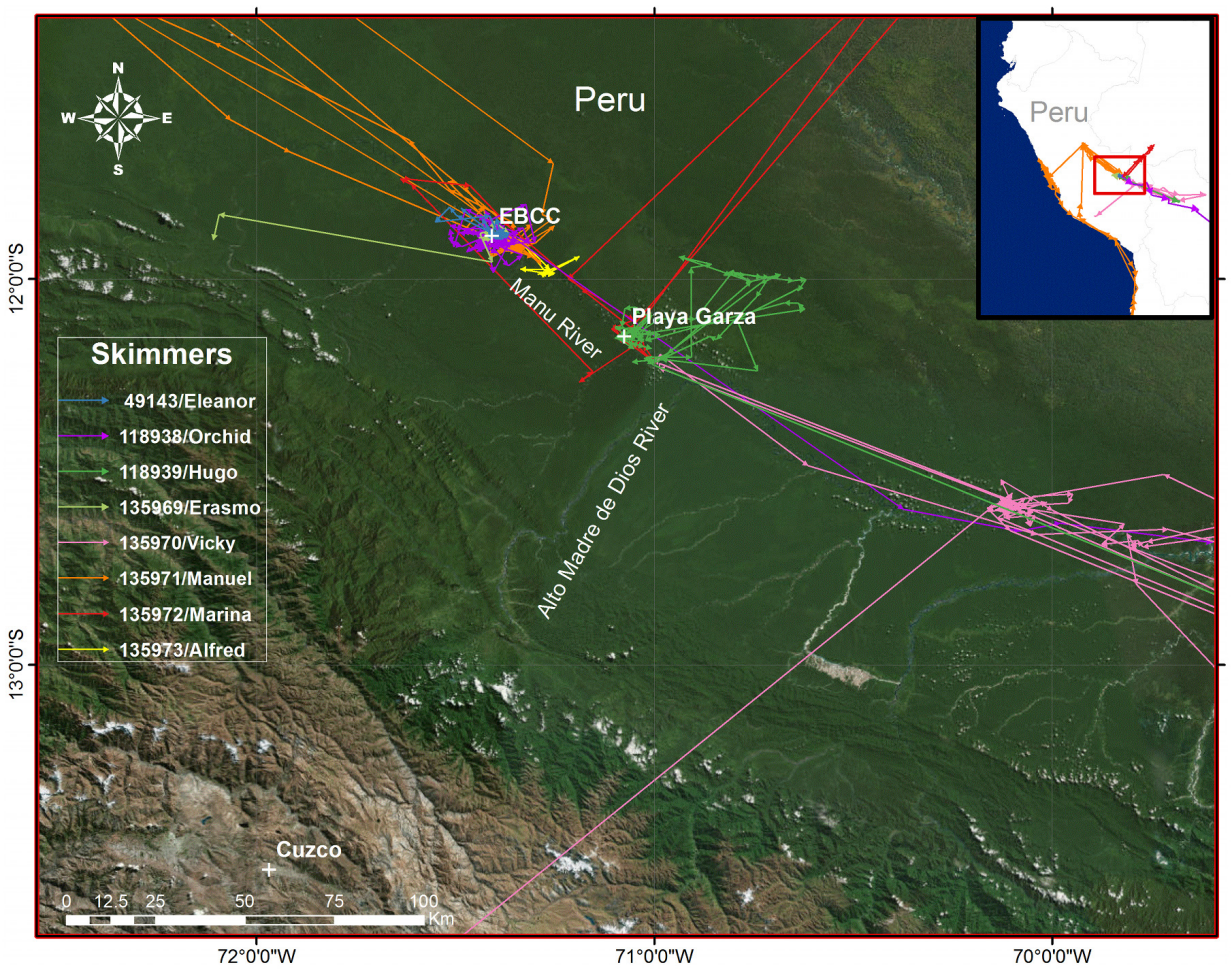
Regional movements of Black Skimmers from Manu National Park. Movements during the breeding season in the region of our two tagging sites: 1) the Cocha Cashu Biological Station (EBCC); and 2) Playa Garza, on the Manu River. Created with ESRI ArcGIS 10.2.2 software. Basemap data sources include: Esri, DigitalGlobe, GeoEye, Earthstar Geographics, CNES/Airbus DS, USDA, USGS, AEX, Getmapping, Aerogrid, IGN, IGP, swisstopo, and the GIS User Community.

We captured a total of 8 Black Skimmers in 2012 and 2014 at two Manu River locations: 1) a nesting beach across from the Cocha Cashu Biological Station (“EBCC”; -11.88° Lat; -71.41° Long), which hosts 4–5 nesting pairs in most years; and, 2) “Playa Garza” (-12.15° Lat; -71.07° Long), a high island in the Manu River approximately 45 km (straight-line distance) downriver of EBCC (Figs 1–3). Playa Garza hosted the largest number of skimmers (32) of all Manu beaches censused between the town of Boca Manu (at the juncture of the Manu and Alto Madre de Dios Rivers) and EBCC in 2014.

We captured skimmers during moonless periods with mistnets (Avinet 6m nets with 127mm mesh) placed alongside nests or at the water’s edge where skimmers forage.

We captured one bird at EBCC in 2012 (August 5), 4 birds at EBCC in 2014 (August 15, 24, 25) and 3 birds at Playa Garza in 2014 (August 16) (Table 1). Each bird was banded with 1–2 colored Darvic legbands, and outfitted with a 5g ARGOS PTT 100 transmitter (Microwave Telemetry, Inc.—MTI) attached using a backpack harness. We used two harness methods. In 2012, the single tagged bird was harnessed using 3/16” Teflon Ribbon (Bally Mills Ribbon) tied with a backpack knotting protocol based on [39]. In 2014, we modified the backpack harness method by eliminating knots on the sternum and crossing the front ribbons through a Neoprene pad through which we cut small slits at each corner for crossing the ribbon (Lisa Ferguson, pers. comm.). The chest pad maintained the ribbons in place with a gap sufficient to space the harness across the wings to prevent constriction of the sternum area and reduction in flight performance.

**Table 1:** Black Skimmer Argos ID’s, deployment statistics and location classes. Number of locations reported are for Full and (Filtered) datasets.

								Number of Locations by Argos Location Class					
Argos ID	Name	Sex	Capture Site	Deployment Date	Working Days	Total # Locations	Distance(km)^1^	3	2	1	0	A	B
49143	Eleanor	F	EBCC	05 Aug 2012	20	53(10)	24	9(4)	16(6)	11(0)	3(0)	4(0)	10(0)
118938	Orchid	F	EBCC	15 Aug 2014	76	142(25)	1982	10(9)	16(7)	37(8)	31(1)	12(0)	36(0)
118939	Hugo	M	Garza	16 Aug 2014	87	133(27)	727	14(11)	18(8)	33(7)	27(1)	13(0)	28(0)
135969	Erasmo	M	EBCC	24 Aug 2014	7	14(2)	1	1(1)	1(1)	4(0)	2(0)	1(0)	5(0)
135970	Vicky	F	Garza	16 Aug 2014	53	129(24)	4833	6(6)	12(7)	32(9)	38(2)	10(0)	31(0)
135971	Manuel	M	EBCC	25 Aug 2014	307	878(135)	12902	15(14)	131(79)	258(41)	221(1)	81(0)	172(0)
135972	Marina	F	Garza	16 Aug 2014	21	60(11)	1158	3(1)	8(4)	13(6)	14(0)	8(0)	14(0)
135973	Alfred	M	EBCC	25 Aug 2014	12	34(6)	11	1(1)	8(4)	6(0)	5(1)	6(0)	8(0)
					Totals:	1443(240)		59(45)	210(115)	394(77)	341(7)	135(0)	304(0)

^1^Total distance traveled between all points in Filtered dataset (one high quality location per duty cycle).

### Ethics Statement

All methods (and LCD fieldwork) were approved under Duke IACUC protocols A081-10-03 and A125-13-05. All work was also undertaken under formal agreement with the Facultad de Medicina Veterinaria y Zootecnia of the Universidad Peruana Cayetao Heredia (UPCH). Wildlife veterinarians (Inés Nole, DVM and Marcos Maguiña, DVM) advised on methods and oversaw fieldwork. Transmitter weights were below the 3% body weight recommended as best practice by the USGS Bird Banding Laboratory [40]. Permission to conduct the work inside Manu National Park was authorized by Perú’s Servicio Nacional de Áreas Naturales Protegidas (SERNANP) under permits SERNANP-PNM-JEF P #13–2012, and #10–2014.

### Data Preparation and Analysis

The PTTs provided data on a duty cycle of 10 h on and 48h off. Data were filtered by Argos using the Kalman Filter [41] and archived at www.movebank.org [42]. ARGOS location classes (“LC”) vary in precision, with LC 3, 2 and 1 locations considered high quality locations, and LC 0, A and B low quality, in descending order of precision ([41] but see [43]). LC Z points are considered invalid. Prior to analyzing data, we applied the Movebank Douglas Argos Filter in DAR mode with the default options [44] to the original 1803 locations. The filter removes all LC Z points and all LC 0, A and B points that suggest unrealistic movement speed and turning angles. In addition, we removed all duplicate timestamps manually, prioritizing higher LC class (1499 points remaining). In some individuals’ final few duty cycles where we continued to receive points, but saw abnormally low movement distances, we analyzed if a subject’s movement radius extended beyond the local average of LC class 3 lat/long coordinates than could be explained by those locations’ error estimates. No birds’ datasets were truncated solely by this criteria, however. In the case of 118938/Orchid, after October 31^st^, about 2 weeks of expected data failed to arrive, and subsequent locations were dominated by LC A and B points, making determination of a local “average” LC 3 reference impractical. We truncated her dataset at the October 31 cutoff, leaving a total of 1443 points in the full dataset (Table 1; Figs 1–3).

For calculations of total distances traveled by individual birds (Table 1), we assembled a truncated dataset using a highly conservative procedure to choose a single high quality location per duty cycle. Duty cycles with no high quality locations were excluded. The process minimizes the calculation of distance traveled by removing the effects of local movements during a duty cycle, and by removing any contribution from errors of precision between points in a duty cycle. The individual point chosen to represent each duty cycle was based on a process similar to the daily “Pick One” function of the Douglas Filter [44] in Movebank editing functions [45]. Points were selected first by comparing location class, secondly by comparing Argos “IQ” quality indicator, and thirdly by comparing the number of messages received by Argos for that location. Filtering, mapping and distance calculations were completed using Movebank’s Data Editing functions [45], ArcGIS 10.2.2 [46] and ArcMET 10.2.2 v3 for ArcGIS [47] respectively.

## Results

### Migration Data Overview

Of eight Black Skimmers tracked with satellite telemetry in 2012 and 2014 (range of 7 to 307 working days), three provided at least partial outbound migration routes (Table 1 and Fig 1) and one of the three (135971/Manuel) completed a full round-trip migration back to the EBCC tagging site. Manuel and one other bird (135970/Vicky) undertook a migration route across the high Andean Cordillera to the Pacific Coast of Perú. Manuel was the first bird to cross the Andes, leaving the Amazonian lowlands between September 24 2229 h and September 26 2149h. Vicky crossed shortly after, between October 6 0905 h and October 8 0822 h, with a final point on October 8 1413h, apparently near Lake Parinacochas (LC Class 0). Eventually, Manuel migrated down the Pacific Coast to the Gulf of Arauco, Chile, arriving there on January 15, 2015, and remaining near there until initiating his return trip on April 10, 2015.

The third long-distance migrant (118938/Orchid) demonstrated an unexpected southeastern migration, traveling over 1800 km from the Manu River to the Paraguay River in eastern Paraguay before transmissions ended. Her departure of September 19 came about one week after the Manu River rose on September 13–16, covering nesting beaches. Orchid arrived in Paraguay by September 24, where she remained for a few weeks before transmissions degraded and ended.

### Local Field Observations

The single bird tagged in 2012 (49143/Eleanor) nested at EBCC and provided only local movements along the Manu River before transmissions ended after 21 days. In 2014, she was re-sighted at EBCC on the beach where she was first tagged, no longer carrying a transmitter but identified by colored leg-bands. She was the only nesting female observed to successfully raise young at the EBCC beach in 2014, suggesting site fidelity for this individual.

The two trans-Andes migrants, Manuel and Vicky, were tagged at the two different capture sites along the Manu River in 2014 and both migrated well before birds were observed to fledge young at EBCC. For example, Eleanor and other pairs with young were observed to remain in the EBCC area until December 2014 (A. Guerra, pers. comm).

Prior to tagging, we did not know whether the birds were nesting or non-reproductive, but local observations provided some subsequent information. Manuel may not have been nesting. After capture he was observed on two occasions (Aug 26 and Sept 24) on a beach several curves downriver of the EBCC tagging site (lat 11°54’ 58” S; long 71° 20’32” W), and only ever observed alone. Vicky was not observed in Manu after tagging. After capturing both Orchid and 135969/Erasmo at EBCC, we observed that these two constituted a pair, nesting together on the EBCC beach. Erasmo, however, was observed (by KG) to be predated by a Roadside Hawk (*Buteo magnirostris*) on September 1 while sitting on the nest.

### Andean Crossings

Although all three Andean crossings occurred in southern Perú, the two trans-Andes migrants appeared to use quite different routes to the Pacific Coast (Fig 2). On his outbound crossing, Manuel moved northwest from the Manu River into the Ucayali drainage before crossing to Isla San Lorenzo offshore of Lima. Vicky appeared to cross the Andes directly from areas she frequented in the lower Madre de Dios River (Perú), arriving approximately 3.8° south of where Manuel arrived. No intermediate points within the Andes were obtained from Manuel’s outbound trip, but his start and end locations suggest he could have used passes near Ticlio (4818m) or Abra de la Viuda (4817m) in a narrow section of the Andes. From Vicky we received one coastal location (although LC A, and filtered by the Douglas filter) and three Andean locations (including 2 Class 0 points and 1 Class B point) prior to her last transmission on October 8. Her Andean locations indicate that she crossed over one of the widest sections of the Peruvian Altiplano in southern Perú (Fig 2).

Manuel’s return migration north along the Chilean coast began on April 10, 2015. He arrived on the Manu River on May 28^th^ after stopovers at the coastal town of Camaná and on the Ucayali River east of the Andes. On the Ucayali, he used some of the same beaches visited on his outbound migration. Details of his second Andean crossing were captured during the PTT’s 10 h duty cycle, providing some high-quality locations along the coast and during the initial hours of ascent. The data showed that Manuel crossed on the night of April 30, leaving the coast between 2131h and 2213 h from a coastal region ~100 km distant from the final locations provided by Vicky on her outbound migration. The data show that Manuel crossed several Andean stream valleys traveling due north before that duty cycle completed on May 1 0155 h.

### Regional Movements

Apart from migratory movements, several of the birds undertook long-distance movements between the Manu River and remote watersheds, some up to >700 km distant. Such long-distance movements were common even during the breeding season, and in several cases involved multiple round-trip visits. Three birds visited the Llanos de Moxos savanna/wetland complex of northern Bolivia (Dept. of Bení) (Fig 3). 118939/Hugo commuted to the upper Río de los Amigos River (Perú) 4 times in one month, and later traveled to the Llanos de Moxos (Bolivia) between September 17 and 19 before transmissions ended there. 135972/Marina twice traveled north to the Río Iaco in Brazil during the 21days her transmitter functioned. Vicky flew to the Llanos de Moxos (Bolivia) and returned to the Madre de Dios River (Perú) 5 times before her trans-Andes migration.

Manuel best demonstrated the long-distance movements of Black Skimmers undertaken off the breeding grounds (Fig 1). After migrating to Isla San Lorenzo (near Lima), Manuel traveled much of the Peruvian coastline, including a foray to ~ 200 km north of Lima (to latitude 9.3° South). He then returned to Isla San Lorenzo before continuing south into Central Chile, ultimately reaching the Gulf of Arauco near Concepción (latitude 37.2° South) where he remained through the austral summer.

## Discussion

Our study is the first to document the varied seasonal movements and migratory behavior of Black Skimmers nesting in Amazonia, and is the first to document a trans-Andean migration route by any Amazonian breeding bird, confirmed by tracking three passages over the high Peruvian Andes by two different birds. The only other documented trans-Andes passage apart from raptors (e.g. condors) is for Arctic Terns (*Sterna paradisaea)* that migrate from the high Arctic to southern oceans through the Southern Andes [35], where the passes are lower (1,500–2,000m) than those used by Peruvian skimmers (nearly 5,000 m). A number of unexpected features of the Peruvian skimmer movements were uncovered by this study, illustrating how little we know about movement patterns of bird populations within South America. Our results suggest that many skimmers observed on the Pacific coast each year may be breeding birds from Amazonia, and that long-distance and/or trans-Andean migration may be more widespread than generally thought for South American birds, including for waterbirds.

The movement of skimmers from freshwater breeding locations towards coastal locales indicates an inter-habitat migration, in which at least some Black Skimmers breeding inland switch from freshwater rivers to rich coastal waters during the non-breeding season. Short distance inter-habitat migrations are known from tropical regions [22,48] but most demonstrations of habitat-switching migrations have been among terrestrial species [9]. In South America, piscivores such as skimmers and terns can access productive marine fisheries along both coasts, including the upwelling zone of the Humboldt Current on the Pacific used by a few of our birds, as well as at the mouths of the Amazon and the Río de la Plata on the Atlantic. Whether inter-habitat migration for Amazonian skimmers is general remains an open question, due to the small number of birds tagged to date. Some individuals tracked in this study migrated up to three months earlier than others in the same population that were observed to rear young. Early migration might suggest a preference for marine feeding grounds, and further might suggest that inland rivers are attractive principally for nest-site characteristics (discussed further below). Regardless, at this stage, we cannot rule out that some birds may migrate elsewhere in other freshwater systems at the start of the high water season on the Manu to find low-water conditions and more concentrated prey.

In addition to high altitude migration through the Andes, we also documented variable directionality in regional and migratory movements, as well as a near-continental scale of recorded migrations, both in latitudinal and longitudinal changes. The unexpected southeastern migration of Orchid through the Llanos de Moxos, Bolivia and into eastern Paraguay suggests there may be several migration routes taken by individual skimmers from our field site. However, only additional tracking would determine how common this route is versus the trans-Andes route or others. Near the end of Orchid’s transmissions, she entered the upper regions of the Paraná Basin where *R*.*n*. *cinerascens* is rarely recorded. Given the ease with which Black Skimmers regularly moved long distances, and the many months taken by Manuel to reach his final austral summer destination, it is conceivable that Orchid’s partial dataset suggests an alternative migration route for some Peruvian skimmers all the way to the Atlantic Coast. Clearly, we do not know if Orchid continued further downriver after transmissions ended. However, had Orchid followed the Paraguay River downstream to the Atlantic Coast, she would have arrived close to the coastal lagoon of Mar Chiquita (latitude -37.7°; longitude -57.4°), a site known to host significant non-breeding concentrations of Black Skimmers, primarily *R*. *n*. *intercedens* [33,49]. We speculate that such cross-continental migrations likely await discovery for skimmers.

Recently, Mariano-Jelicich & Madrid [33] found that *R*. *n*. *intercedens* in Mar Chiquita are not panmictic, but have low genetic differentiation, which they interpret as a consequence of mixing far-flung breeding populations during the non-breeding season. Our results reinforce this interpretation by documenting large-scale movements of individual birds, and raising the possibility that at least some birds from western Amazonian sites join the non-breeding congregations found at mid-latitude Atlantic sites.

Another notable finding of this research was the repeated instances of “commuting” to distant watersheds even during the breeding season. All birds tracked for more than 30 days traveled to a remote watershed at least once, and covered at least 600 km (Table 1). While breeding season travel was unexpected, we interpret the data as movement occurring in between nesting attempts or after total reproductive failure for the year (i.e. Orchid). The Manu system is particularly close to the Andes and therefore intermittently influenced by regional precipitation, making unpredictable flooding of nests a periodic cause of coincident nest failures. Skimmers place nests surprisingly close to the water’s edge, presumably to aid thermal regulation of nests on cooler sand while also allowing better detection of predators [50], but by doing so, the risk of flooding is high and is regularly observed in Manu.

Within South American migrant birds, skimmers are not alone in moving long distances, although they may be unusual in doing so extensively within the breeding season. Jahn et al [12] documented long-distance movements of Fork-tailed Flycatchers (*Tyrannus savana*) using geolocators, documenting the largest latitudinal shift for any individual bird within South America to date, approximately 4100 km distance on fall migration. The authors suggest that long-distance migration within South America may be more common than previously thought, which our data definitely support. Even some Neartic-Neotropical migrants move considerable distances between regions of South America during the non-breeding season, as shown by the intra-tropical movements of Veeries (*Catharus fuscescens*) during their non-breeding season [14]. In Australia, nomadic Banded Stilts (*Cladorhynchus leucocephalus*) have been shown to move across the continent in response to rainfall events, connecting populations previously thought distinct [16].

A final distinctive feature of our findings is the observation that skimmers breeding in the tropical Amazon migrate south to spend the non-breeding period at temperate latitudes. This is the reverse of the pattern followed by the great majority of austral migrants that breed in summer at temperate latitudes and migrate to the tropics for the austral winter. In contrast, Black Skimmers breeding in coastal North America migrate to lower latitudes for the winter [51]. The pattern displayed by Peruvian skimmers is also directly countercurrent to that shown by African Skimmers that breed just within the tropical zone of southern Africa (i.e. in the Okavango and Zambezi systems) and migrate north, to lower latitude sites, for the austral winter [25]. Peruvian skimmers are also anomalous by breeding in the austral winter, while sitting out the austral summer at southern latitudes equivalent to those favored for breeding by North American skimmers in the Northern Hemisphere. While the Peruvian skimmers’ migration therefore broadly tracks the latitudinal movements of other austral migrants temporally, they do so while at cross purposes in terms of reproduction.

Curiously, few other tropical breeding bird species spend the non-breeding season at temperate latitudes, although some African species migrate from non-breeding tropical locations to both northern and southern temperate regions to breed [18]. Why don’t South American skimmers follow the usual pattern of breeding in the austral summer and spending the non-breeding season at lower latitudes? A need to escape high water conditions only partly accounts for the pattern. It must be that the fitness of birds nesting on Amazon beaches is reliably higher than at coastal alternatives. Seasonal fluctuations in coastal productivity may make critical resources unreliable across large areas [52], affecting reproductive success; however, predation risk might also differ between the two options. High populations of both mammalian (e.g. foxes–*Dusicyon* spp. and felines, such as Geoffroy’s cat *Oncifels geoffroyi*) and avian (e.g. Kelp Gulls, *Larus domincanus*) predators that prey on waterbirds and chicks [49,53] are common in potential coastal breeding habitats in temperate South America. Elsewhere, foxes have been shown to impart strong control over seabird colonies [54], and increases in gull and Bald Eagle populations are correlated with ongoing declines in skimmers and other colonial waterbirds breeding in Virginia, USA [55]. While nesting success for passerines is generally low in tropical forests [56], skimmers can benefit from nesting on river islands [57] and from multi-species defense provided by allies, including the Large-Billed Tern (*Phaetusa simplex*) and Yellow-Billed Tern (*Sterna superciliaris*) [29]. The latter two species co-occur across the entire Amazon basin, yet neither appears on the Pacific Coast.

Migratory routes, corridors and stopover sites are poorly known for Amazonian waterbirds, yet learning them is critical for conserving such highly mobile organisms such as waterbirds [10,34,58]. Stopovers used by Manuel for > 2 days along the Pacific coast are few but instructive; each was a location near the mouth of a freshwater river (i.e. Lima, Barranca District, Lagunas de Mejia, and Camaná in Perú plus the Mataquito and Itata Rivers in Chile). Unfortunately, most freshwater lagoons along the Pacific coast of South America are heavily altered for agricultural use, despite their importance to migratory birds [34]. Within the continent, the use of the Llanos de Moxos, Bolivia by multiple skimmers from Peru, both for feeding during the breeding season and as a possible migration corridor to the southeast, is a potentially significant finding relative to current conservation issues in the region. The Llanos de Moxos is the wet season destination for the critically threatened Peruvian population of Orinoco Geese (*Neochen jubata*) and for Orinoco Geese from the Río Juruá, Brazil ([23] and Davenport, Endo and Peres unpubl. data). The Bolivian Andes-Amazon region is also important for multiple terrestrial austral migrants [9]. Despite being recently declared a Ramsar site, very little of the Llanos de Moxos enjoys strict protection with ~ 98% of the land area still in private hands (B. Hennessey, pers. comm.). Development plans for the Llanos de Moxos and the Andes-Amazon region include extensive activities that will disrupt wetland connectivity and function including agricultural intensification (e.g. sugar cane, cattle), dam-building, and mining and forestry concessions [10,59–61]. Dams are a particular concern for waterbirds, especially for skimmers that require shallow, slow-moving water for feeding and intact disturbance regimes to maintain beach dynamics. In the Zambezi River, numbers of breeding African Skimmers have declined since the construction of Kariba Dam and the regulation of the downstream flooding regime and consequent changes to river dynamics and vegetation dynamics on beaches [25].

## Conclusion

Overall, our data and interpretations represent only a first step to documenting migratory and movement behavior within Amazonia and applying lessons learned to conservation. We draw attention to the fact that even one small-scale study on a single species has produced so many surprises of unsuspected movement phenomena. The great mobility demonstrated by the skimmers of Manu, both during and outside the breeding season, makes them an interesting case for studying evolutionary strategies of adaptation to the Amazon’s unique situation. More information is clearly needed on Amazonia’s migratory animals, in light of the rapidly expanding threats to the integrity of freshwater ecosystems and the paucity of existing baseline data on animal movements in South America. Even with low sample sizes, satellite telemetry provides important new insights, including documenting new migration patterns for South America. We suggest that additional research on movement patterns of Amazonian waterbirds should be guided by an appreciation of the opportunities offered to migratory birds at the scale of the entire Amazon Basin with its complex, interconnected and interdependent flood-pulse ecology [62].
